# Carbon Material Based Electrochemical Immunosensor for Gastric Cancer Markers Detection

**DOI:** 10.3389/fchem.2021.736271

**Published:** 2021-08-31

**Authors:** Zhuliang Zhang, Minsi Peng, Defeng Li, Jun Yao, Yingxue Li, Benhua Wu, Lisheng Wang, Zhenglei Xu

**Affiliations:** Department of Gastroenterology, Shenzhen People’s Hospital, The Second Clinical Medical College, Jinan University, Shenzhen, China

**Keywords:** carbon materials, immunosensor, carbon nanotubes, graphene, gastric cancer, biomarker

## Abstract

Gastric cancer is one of the most common malignant tumors, and early diagnosis will be of great significance to improve the survival quality and overall treatment outcome evaluation of patients. Nanoelectrochemical immunosensor is an emerging biosensor combining nanotechnology, electrochemical analysis method and immunological technology, which has simple operation, fast analysis speed, high sensitivity, and good selectivity. This mini-review summarized immunoassay techniques, nanotechnology and electrochemical sensing for the early detection of gastric cancer. In particular, we focus on the tension of carbon nanomaterials in this field, including the functionalized preparation of materials, signal enhancement and the construction of novel sensing interfaces. Currently, various tumor markers are being developed, but the more recognized gastric cancer tumor markers are carcinoembryonic antigen (CEA), carbohydrate antigen (CA), CD44V9, miRNAs, and programmed death ligand 1. Among them, the electrochemical immunosensor allows the detection of CEA, CA, and miRNAs. The mini-review focused on the development of using carbon based materials, especially carbon nanotubes and graphene for immunosensor fabrication and gastric cancer markers detection.

## Introduction

In recent years, with the increase in the number of gastric cancer patients and their mortality rate, the methods of gastric cancer diagnosis are in urgent need of solution. From the large number of patients who died of gastric cancer, it can be seen that the main reason for the death is the late detection and untimely treatment. In order to better prevention and detection, early gastric cancer detection is crucial. The current methods of early gastric cancer detection include gastroscopy, radioactivity (Archer and Grant, 1991), ultrasound, serum gastric function test, and gastrin 17 combined with pepsinogen serological test. These assays have limitations, such as gastroscopy, ultrasound, and radiological testing can be uncomfortable for patients and are not particularly effective. Biologic tumor markers for gastric cancer is a more common and hot research direction. It can be detected quickly and without negative effects on the patients themselves. Currently, various new tumor markers are being developed, but the more recognized gastric cancer tumor markers are carcinoembryonic antigen (CEA) (Ishigami et al., 2001; Zhang et al., 2021), carbohydrate antigen (CA) (Gaspar et al., 2001), CD44V9 (Mashima et al., 2019), miRNAs (Khandelwal et al., 2019), and programmed death ligand 1 (PD-L1) (Wang et al., 2019).

Biosensors are a multidisciplinary intersection that includes medical, biological, chemical, and electronic technologies (Karimi-Maleh et al., 2021a; Luo et al., 2021). The principle of biosensor analysis and detection is that when the substance to be measured specifically binds to a molecular recognition element (e.g., enzyme or antibody, etc.), the resulting complex is transformed by a converter into a signal that can be output, such as an optical signal or an electrical signal. Electrochemical biosensors are divided into current, potential, capacitance, and conductivity types according to the measurement signal. According to the category of bioactive molecules, they can be divided into enzyme sensors, tissue sensors, immunosensors, DNA sensors, and microbial sensors. Among them, electrochemical biosensors for monitoring specific reactions between antigen˗antibodies are called electrochemical immunosensors. There are two key directions in the research of electrochemical immunosensors: 1) designing novel biocompatible biocomponent immobilization interfaces to improve the loading and bioactivity of biocomponents on the sensing interface; 2) employing novel immunoreactive signal amplification techniques to enhance the sensitivity of the sensors. Therefore, there has been a search for good materials for immobilization of bioactive substances and efficient methods for immobilization of biological components.

Nanomaterials have become one of the popular materials for research and application in recent years due to their unique properties in optical, magnetic, electrical, and catalytic properties (Medetalibeyoğlu et al., 2020; Akça et al., 2021; Karimi-Maleh et al., 2021c; Liu et al., 2021). As a modifying material for sensor interfaces and as a solid-loading matrix for biorecognition molecules. Due to their large specific surface area, high surface free energy, good biocompatibility, and rich in surface functional groups, nanomaterials can be used in the construction of biosensing interfaces to effectively increase the specific surface area of electrodes, increase the loading of biomolecules on the electrode surface, accelerate the electron transfer rate, enhance the conductivity of modified electrodes, and accelerate the response of sensors. Carbon is a widely studied and used material in electrochemical sensor preparation. In the macroscopic field, there are three forms of carbon isomers: graphite, diamond, and amorphous carbon. However, the discovery of fullerenes (C60) and carbon nanotubes (CNTs) has led to new forms of carbon materials, and since then, the research on carbon nanomaterials has been booming. In this mini-review, we review the application of carbon nanomaterials, especially CNTs and graphene, in the preparation of electrochemical immunosensors and in the detection of gastric cancer biomarkers.

### Carbon Materials Based Electrochemical Biosensors

Carbon nanomaterials play an important role in bioelectrocatalytic reactions due to their high specific surface area and their better biocompatibility (Naderi Asrami et al., 2020; Karaman et al., 2021; Karimi-Maleh et al., 2021b). Their large specific surface area and high surface reactivity lead to enhanced adsorption capacity of the materials, increased active sites on the surface, and improved catalytic efficiency. Compared with conventional sensors, carbon nanomaterials sensors are not only smaller and faster, but also more accurate and reliable. the discovery of CNTs has largely enriched the research of carbon materials and triggered a *trans*-generational material revolution. Britto et al. (1996) first made CNTs into electrodes and used them for the electrocatalytic oxidation of the neurotransmitter dopamine. The preparation method was similar to that of carbon paste electrodes, using bromine imitation as the binder. The CNTs have a good catalytic effect on the electrochemical oxidation of dopamine. Timur et al. (2007) reported the preparation of microbial sensors generated by immobilizing *Pseudomonas aeruginosa* cells on CNTs with osmium polymers as intermediates. By optimizing the amount of CNTs and polymers, the electron migration rate can be increased and can be used for microbial fuel cells and biochemical oxygen demand (BOD) measurements in wastewater.

CNTs have high axial strength and stiffness, so they are often used as reinforcement for composites. Meanwhile, their excellent electrical and thermal conductivity can improve the functionality of composite materials. The use of conducting polymers as sensing electrodes has attracted much attention due to their excellent electrochemical properties. The CNTs/polymer composite materials can be divided into two categories. The first one is to use CNTs as the main body and modify the polymer on the wall of CNTs to increase the solubility of CNTs. Another category is based on polymers as the main body and CNTs as filler materials, mainly for conductive polymer materials, with the aim of improving the conductivity and stability of conductive polymers. Commonly used conductive polymers such as polyaniline (PANI) and polypyrrole (PPy) (Pei et al., 2019; Assari et al., 2020). They have good electrochemical properties to amplify the signal current and eliminate the influence of electrode impurities, and provide a suitable environment for immobilizing biomolecules. Not only the complexation of conductive polymers with CNTs, but also the complexation of noble metals with CNTs has attracted a lot of attention from researchers. Among many noble metals, AuNPs have attracted much attention from researchers due to their ability to maintain the biological activity of adsorbed enzymes and reduce the hindrance of direct electron migration by protein shells.

In addition to CNTs, carbon nanofibers (CNFs) have similar high electrical conductivity, high mechanical strength, large specific surface area, and thermal stability as CNTs. Both of them can be used as good metal nanoparticle catalyst carriers with promising applications in chemical/biological sensing and catalysis. Sheng et al. (2010) introduced porous carbon nanofiber (PCNF)/room temperature ionic liquid (RTIL) membranes, which provide a suitable environment for direct electron migration of ferrous hemoglobin proteins. Hemoglobin (Hb), myoglobin (Mb), and cytochrome C showed a pair of reversible redox peaks on the PCNF/RTIL-modified membranes.

Graphene has received great attention in the field of biosensors in the last decade due to its high specific surface area, fast electron transfer capability and good biocompatibility. Huang et al. (2011) prepared a sensitive label-free electrochemical immunosensor by modifying carbon ionic liquid electrodes with amino-functionalized graphene and AuNPs complexes. They first applied the amino-modified graphene dropwise on the surface of the carbon paste electrode, and then used electrostatic interaction to adsorb a layer of negatively charged AuNPs on the surface of the GO. A layer of antibodies. The modified electrode is then immersed in bovine serum albumin (BSA) solution to block the active site for non-specific adsorption. By the specific interaction of anti-AFP on AFP, this sensor can detect different concentrations of AFP.

### Carcinoembryonic Antigen Detection

CEA, an acidic glycoprotein with human embryonic antigenic properties, was first discovered in 1965. Chen et al. (2012) found that perioperative chemotherapy improved overall survival in patients with normal CEA levels prior to treatment in 2012. Bacac et al. (2016) discovered a novel bispecific antibody to carcinoembryonic antigen T cells for the treatment of CEA-expressing solid tumors currently in phase I clinical trials in 2016.

CNTs are a very useful substrate material. For example, Lv et al. (2018) immobilized bimetallic core-shell rhodium@palladium nanodendrites (Rh@Pd NDs) onto sulfate-based functionalized MWCNTs. This composite can be used as a simple electrochemical immunosensor. Rh@Pd NDs possess a unique dendritic nanostructure that provides an abundance of catalytically active sites. Meanwhile, MWCNTs improve the performance of the sensor due to their excellent electrical conductivity, good solubility, and high surface area. Their proposed electrochemical immunosensor can provide linear detection of CEA within 25 fg/ml-100 ng/ml under optimal conditions. To further improve the immobilization performance, cyclodextrins can be modified on CNTs. Han et al. (2017) prepared a novel sensitive sandwich-type non-enzymatic electrochemical immunosensor using cyclodextrin-modified MWCNTs. Synthesized silver nanoparticles-carbon nanotubes/manganese dioxide (Ag NPs-MWCNTs/MnO_2_) were used as a label for Ab2 to achieve duplex amplification of the electrochemical signal. This immunosensor can reach the detection limit of 0.03 pg/ml for CEA. In addition to cyclodextrins, chitosan also has a similar role. Gao et al. (2011) prepared a chitosan-carbon nanotube-gold nanoparticles nanocomposite membrane. This composite membrane was able to exhibit better electrical conductivity, high stability and good biocompatibility due to its three-dimensional structure. Under optimal conditions, this immunosensor can detect CEA in two linear ranges between 0.1–2.0 and 2.0–200.0 ng/ml with a detection limit of 0.04 ng/ml.

Graphene is now replacing carbon nanotubes in the development of many sensors. For example, Jozghorbani et al. (2021) developed a label-free electrochemical immunosensor for the detection of CEA. They modified reduced graphene oxide (rGO) on the surface of a glassy carbon electrode to form an interface for binding antibodies. rGO has carboxyl groups on the surface that can be used to bind to antibodies. This binding between antibody and rGO can be confirmed by CV and EIS characterization. The weakening of the electrochemical signal can be used as a signal of successful antibody modification. This immunosensor allows linear detection of CEA in the concentration range of 0.1–5 ng/ml. The detection limit can reach 0.05 ng/ml. Chen et al. (2018) prepared AuNPs-TiO_2_-graphene composites for sandwich immunosensing detection of CEA. in this work, graphene was first modified with dopamine via π-stacking and then immobilized with TiO_2_ nanoparticles. After that, AuNPs-TiO_2_-graphene composites were synthesized by photoreduction method under UV irradiation. The AuNPs interacted with HRP-Ab2 and covalently attached HRP-Ab2. Under optimal conditions, this sandwich immunosensor could detect CEA linearly in a wide range of 0.005–200 ng/ml, and the detection limit It can reach 3.33 pg/ml.

### Carbohydrate Antigen Detection

CA is a tumor cell-associated antigen. CA125, CA19-9, and CA72-4 were considered to have the strongest correlation with gastric cancer. Clinical data from Abbas et al. (Gao et al., 2018) showed that elevated pre-treatment CA199 levels are associated with a higher risk of tumor progression and a worse prognosis in gastric cancer. CA199 can be reduced with the use of anti-angiogenic agents and first-line platinum-based chemotherapy.

The role of carbon material in the CA199 assay is very similar to that of the previous assay for CEA. Kalyani et al. (2021) reported an electrochemical immunosensor based on MWCNT-Fe_3_O_4_, which was dispersed in chitosan for immobilization of specific antibodies. The detection range of this electrochemical immunosensor was 1.0 pg/ML-100 ng, ml. the detection limit was 0.163 pg/ml. Very similarly, Huang et al. (2017) synthesized a polysulfate-gold composite (AuNPs@PThi). This AuNPs@PThi can act as a sensitive redox probe and is capable of amplifying electrochemical signals. Under optimal conditions, this label-free immunosensor can linearly detect CA199 from 6.5–520 U/ml with detection limits up to 0.26 U/ml.

**FIGURE 1:**
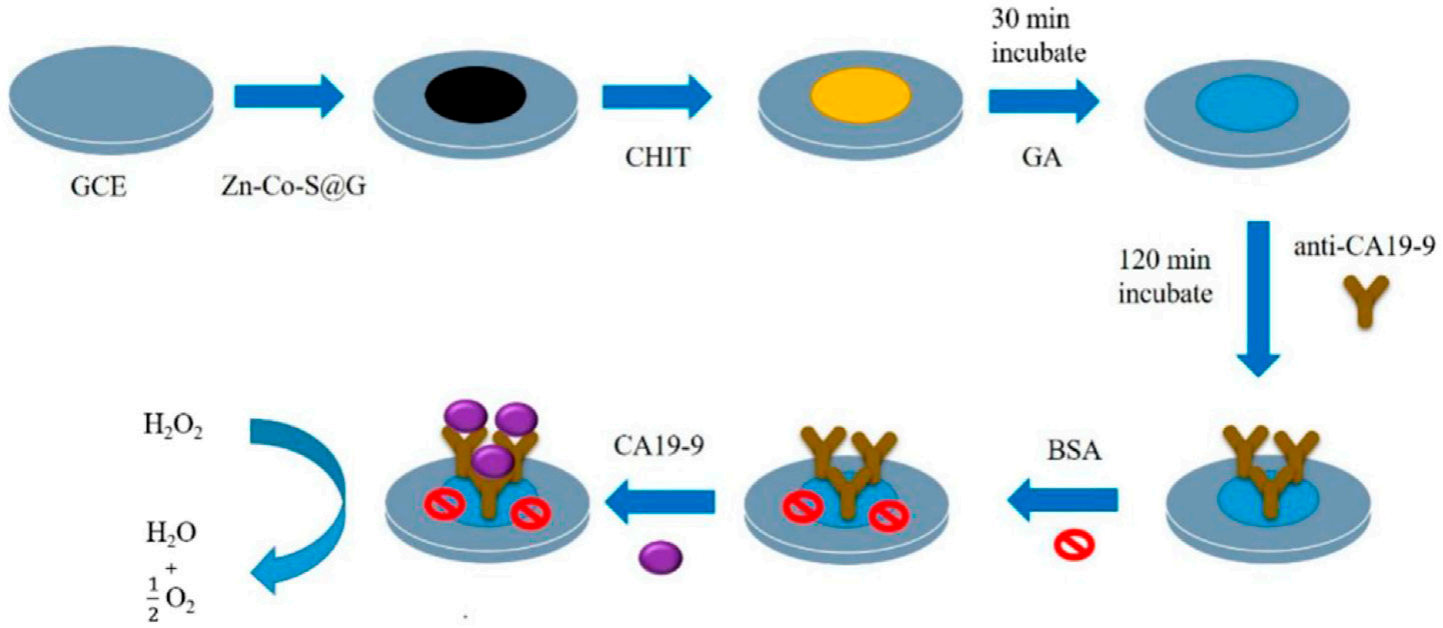
Scheme of fabrication of the Zn-Co-S/graphene electrochemical immunosensor for CA199 detection.

The function of graphene here also remains as a substrate and is used to improve the performance of the electrodes. As shown in Figure 1, the Zn-Co-S/graphene composite can be used to detect CA199 (Su et al., 2021). The Zn-Co-S dot-like nanoparticles grown on graphene can form a conductive network, allowing the direct reduction of H_2_O_2_ by the active site. This electrochemical immunosensor can detect CA199 linearly between 6.3 and 300 U/ml with a detection limit of 0 U/ml. For practical considerations, electrochemical immunosensors can also be assembled on screen-printed electrodes (SPEs). Mic et al. (2020) modified SPEs with thermally reduced graphene oxide (TRGO). The modified electrodes can interact with CA199-His molecules by adsorption. It was shown that TRGO has a very strong affinity for CA199-His, to the extent that it can be used for quantitative detection.

### miRNA Detection

miRNAs are a class of small non-coding single-stranded RNAs about 19–25 nt long that are commonly found in plants and animals. They can inhibit the transcription or translation of target genes by incomplete binding to target mRNAs to perform their biological functions. Since the year 2,000, miRNAs have been widely studied worldwide and more than two thousand miRNAs have been identified and characterized. miRNAs are widely involved in the development of gastric cancer (Daneshpour et al., 2018; Huang et al., 2019). *H. pylori* (HP) is a class I gastric carcinogen. miR-223, miR-125a, and miR-21 expression was found to be significantly increased in positive gastric cancer specimens, while miR-218 expression was significantly decreased. This suggests that HP infection may promote gastric cancer formation by altering the corresponding miRNAs. Several studies also demonstrated that miR-551b-3p, miR-100-5p, and miR-363-3 were significantly down-regulated in gastric cancer tissues and cells. miR-215 was significantly up-regulated in gastric cancer tissues and cells. miR-429 aberrant expression may be associated with age differences in gastric cancer development. However, there are not many applications of carbon nanomaterials in miRNA detection. Yammouri et al. (2017) compared the detection of miR-125a by carbon black, MWCNT and GO after preparing an electrochemical immunosensor. Surprisingly, the lowest detection limit of microRNA-125a was obtained with the sensor modified with carbon black.

## Conclusion

Gastric cancer is a malignant tumor originating from the epithelial cells of the gastric mucosa, and it is the second leading cause of cancer death worldwide. The development of new immunoassay techniques that are rapid, sensitive, high-throughput, low-cost, easy for clinical dissemination and field application is crucial for early diagnosis, prognosis monitoring, and treatment of gastric cancer. Carbon nanomaterials have very high specific surface area, electrical conductivity and good mechanical properties, which are ideal materials required in electrochemistry. A large number of theoretical and practical studies have been conducted on the application of carbon nanotubes and graphene in the field of electrochemistry, which fully demonstrate the application prospects of carbon materials as new sensing materials. An ideal tumor marker should have high sensitivity, good specificity, and high accuracy. Although many tumor markers have been continuously discovered, studied and applied, a tumor marker with high sensitivity, and specificity has not been found yet. Immunosensors will become a widely used novel test in the field of medical diagnosis. Various novel signal-enhanced immunosensors can effectively improve the sensitivity and specificity of tumor marker diagnosis and enable the detection of gastric cancer markers.
